# Anisotropic functionalization of upconversion nanoparticles†
†Electronic supplementary information (ESI) available. See DOI: 10.1039/c8sc01023d


**DOI:** 10.1039/c8sc01023d

**Published:** 2018-04-23

**Authors:** Wei Ren, Shihui Wen, Sherif Abdulkader Tawfik, Qian P. Su, Gungun Lin, Lining A. Ju, Michael J. Ford, Harshad Ghodke, Antoine M. van Oijen, Dayong Jin

**Affiliations:** a Institute for Biomedical Materials & Devices (IBMD) , Faculty of Science , University of Technology Sydney , Ultimo NSW 2007 , Australia . Email: dayong.jin@uts.edu.au; b Heart Research Institute , Charles Perkins Centre , The University of Sydney , Camperdown NSW 2006 , Australia; c School of Chemistry , University of Wollongong , Illawarra Health and Medical Research Institute , Wollongong NSW 2522 , Australia

## Abstract

Despite significant advances toward accurate tuning of the size and shape of colloidal nanoparticles, the precise control of the surface chemistry thereof remains a grand challenge. It is desirable to conjugate functional bio-molecules onto the selected facets of nanoparticles owing to the versatile capabilities rendered by the molecules. We report here facet-selective conjugation of DNA molecules onto upconversion nanoparticles *via* ligand competition reaction. Different binding strengths of phosphodiester bonds and phosphate groups on DNA and the surfactant molecules allow one to create heterogeneous bio-chemistry surface for upconversion nanoparticles. The tailored surface properties lead to the formation of distinct self-assembly structures. Our findings provide insight into the interactions between biomolecules and nanoparticles, unveiling the potential of using nanoparticles as fundamental building blocks for creating self-assembled nano-architectures.

## Abstract 2

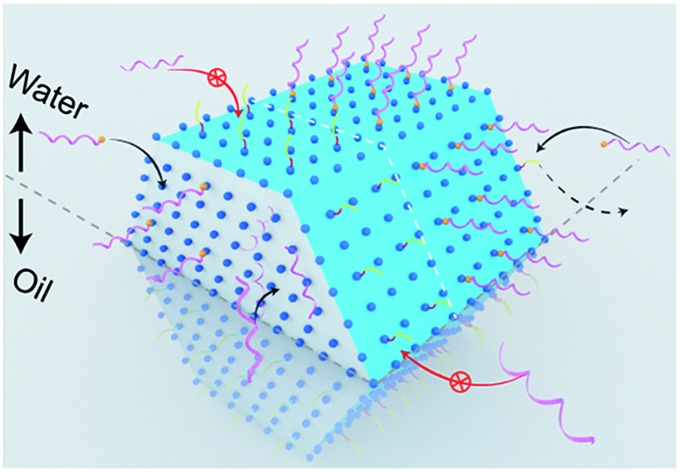
Ligand competition directs heterogeneous bio-chemistry surface and self-assembly for upconversion nanoparticles.

## 

Doped by rare-earth ions, hexagonal-phase (β) NaYF_4_ upconversion nanoparticles (UCNPs) are a new generation of nanomaterials featuring step-wise photon anti-Stokes emission: pumped by near infrared laser to emit visible fluorescence,^1^ tunable lifetime values from tens of microseconds to several hundred microseconds^2^ and low toxicity in biological systems.^3^ Owing to such exceptional optical properties, a diversity of applications have been realized using UCNPs, for instance, background-free biomolecular detection,^4^–^6^ *in vivo* bio-imaging,^7^,^8^ forensic applications,^9^–^11^ anti-counterfeiting applications,^12^–^14^ super resolution imaging,^15^,^16^ and nanoscale thermometry.^17^,^18^ Towards bio-related applications, the key is to functionalize the surface of UCNPs by specific biomolecules modification and transfer them from organic solvent to aqueous phase. Up to now, a variety of strategies have been devoted to modifying the surface of UCNPs, including amphiphilic polymer interaction,^7^,^19^ silica coating,^20^–^22^ surface ligands oxidization^23^ and ligand exchange;^24^,^25^ nevertheless, facet-selective functionalisation of upconversion nanoparticles has seldom been reported.

DNA appears to be one of the most popular biomolecules for surface functionalisation of nanoparticles, due to its commercial availability, low cost, excellent stability and specificity that allows direct recognition of complementary sequences. In 2005, Costa and co-workers reported that the backbone of DNA molecules can bind to the lanthanide ions,^26^ which suggests a new way to directly conjugate DNA onto UCNPs. Based on this finding, researchers have developed a one-step conjugation technique to attach DNA onto the surface of UCNPs;^24^,^25^ nevertheless, such a method still treats UCNPs as spherical nanoparticles and overlooks the fact that UCNPs are hexagonal prism nanoparticles with two (001) facets on the tips and six (100)/(010) facets on the lateral surface, and these facets have different charge distribution and are capped by different ligand molecules.^27^ Hence, we hypothesize that the binding strength of DNA could be varied on the (001) and (100)/(010) facets which may lead to selective molecule binding on the different facets of UCNPs. If this is true, in-depth understanding and proper control of anisotropic surface properties will lead to a new scope for bio-/nano-interface chemistry and applications.

In this paper, we utilize DNA to investigate the facet-selective binding to the surface of UCNPs. We find that the binding affinity of phosphodiester bonds on the backbone of DNA is stronger than oleic acid (OAH) on (001) facets but weaker than oleate anions (OA^–^) on (100)/(010) facets, resulting in selective binding to the two ends of UCNPs; whereas the phosphate group on the end of DNA shows the strongest affinity to replace all the surfactant molecules on UCNPs which creates hydrophilic surface. The location of DNA molecules is experimentally confirmed by analytical chemistry methods and directly visualized by the stochastic optical reconstruction microscopy (STORM). The facet-selective functionalization of UCNPs not only provides insights into the understanding of bio-/nano-interface reaction but also has potential application in self-assembly of structures of nanoparticle building blocks.

## Results and discussion

### DNA ligand exchange and quantification

**Fig. 1:**
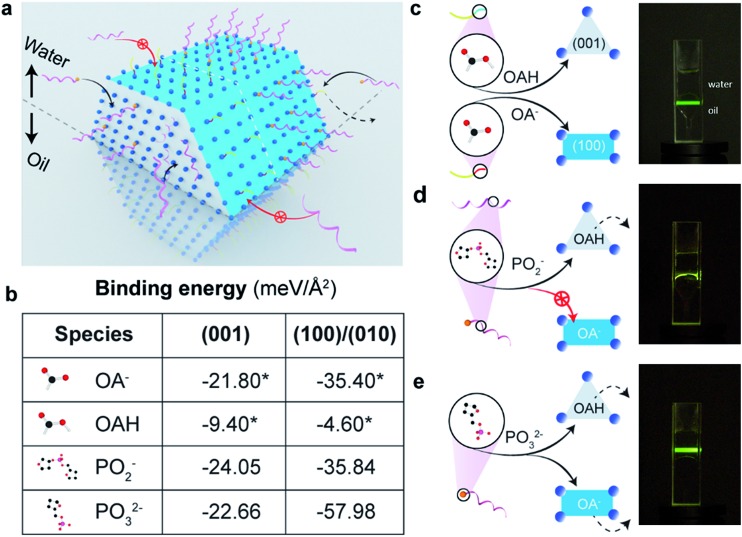
The anisotropic surface properties of rare-earth doped hexagonal-phase upconversion nanocrystals. (a) Illustrative figure shows the ligand competition at the interface of water (upper) and organic solvent (under). Two types of single-strand DNA with (helical pink strand tipped with a yellow sphere) and without (helical pink strand) a phosphate group on the 5′ terminus have been used in the work. (b) Computational simulation results in different binding energies of two kinds of surfactant molecules, OAH and OA^–^, and two types of single DNA strands with and without phosphate groups on the (001) and (100)/(010) facets of the nanocrystal. Remarkably the binding strength of phosphodiester bonds on (100)/(010) facets are not strong enough to replace OA anions but sufficiently strong to replace OAH on (001) facets. Note: the binding energies marked with asterisks are obtained from our previous publication.^27^ (c)–(e) Illustrative figures to show ligand competition on different facets and the location of UCNPs when transferring them from organic phase into upper level of aqueous phase after ligand exchange. (c) Initially, the as-synthesized UCNPs are capped by OAH and OA^–^ and suspended in the organic phase. (d) When the ratio of the amounts of hydrophilic molecules (DNA) to that of hydrophobic surfactants (OA^–^ and OAH) reaches a balance, the nanorods with anisotropic surface properties only stay at the water/oil interface. (e) When modified by phosphate group on the terminus, DNA molecules can replace all the organic surfactant molecules on the surface of UCNPs and pull them up to the upper aqueous phase.

To amplify the anisotropic surface properties of UCNPs, instead of using small nanoparticles with small aspect ratios, we choose nanorods of ∼170 nm in length and ∼35 nm in diameter for the study. The ligand exchange reaction is taken place by mixing the rods suspended in chloroform and DNA aqueous solution followed by gentle shaking for 3 hours. Fig. 1a illustrates the ligand competition process with two kinds of hydrophilic molecules: single strand DNA molecules with and without a phosphate group on the 5′ terminus. The rods are transferred from chloroform into the upper aqueous phase by replacing the surfactants, *i.e.*, oleic acid molecules (OAH) and oleate anions (OA^–^) on the (001) and (100)/(010) facets of the particles, respectively.

We compute the binding energies of four different chelating moieties, *e.g.* oleic acid, oleate anion, phosphate group and phosphodiester bond, onto the two kinds of facets of the hexagonal prism-like UCNPs based on density functional theory (DFT) simulation (Fig. 1b, table). The binding strength of phosphate groups to the surface of UCNPs is remarkably stronger than that of the surfactant molecules, which results in the fact that the phosphorylated DNA can replace the initial surfactant molecules on all the facets of the particles; whereas phosphodiester bonds is not strong enough to compete with OA^–^ on (100)/(010) facets thus only replace the OAH molecules on the (001) facets.

We propose three scenarios of how the different ligands compete to attach to the anisotropic surfaces of UCNPs (Fig. 1c–e), which are experimentally verified from the locations of the UCNPs after the ligand competition and exchange process. After completing reaction with DNA ligands without the 5′-terminus modified with phosphate groups, it is shown that UCNPs featuring a mixture of hydrophilic and hydrophobic surface properties are suspended between chloroform (oil) and water (Fig. 1d). In contrast, by using DNA ligands with 5′ terminus modified with phosphate moiety groups that display the strongest binding to both (100)/(010) and (001) facets, upconversion nanorods are completely pulled into the aqueous suspension (Fig. 1e). Due to the varied surface quenching effects on the green and red emission bands of UCNPs,^28^,^29^ the colour of UCNPs was slightly changed after being transferred from the organic phase to the aqueous phase (Fig. S3†). The degree of anisotropic surface properties can be fine-tuned by decreasing the pH value of DNA solution, which induces more DNA molecules to be bonded onto the side surfaces (100)/(010) facets of the UCNPs, see ESI Section 3.†

### Determine the location of DNA molecules on UCNPs

**Fig. 2:**
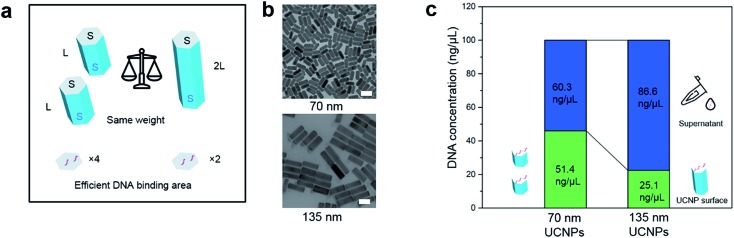
Quantitative verification of single-strand DNA molecules' selective binding to the end surface of nanorods. (a) Design of an analytical experiment using two samples of nanorods at same weight. The length of the longer rods doubles that of the shorter rods. When both samples are at same weight, the sample of the shorter rods has total end-surface areas nearly twice that of the longer rods. (b) TEM images of 70 nm nanorods and 135 nm nanorods. In a ligand exchange experiment, if DNA molecules only bind to the end surface, the sample of shorter rods should absorb nearly twice the amount of DNA molecules than the longer rods. (c) By checking the amount of residue DNA molecules in suspension, the amount of DNA molecules being capped onto the nanocrystals are quantitatively compared. Scale bar: 100 nm.

To quantitatively evaluate the selective binding of single strand synthetic DNA ligands, we design and synthesize two types of nanorods with different length (∼70 and ∼135 nm, TEM images shown in Fig. 2b), and conduct a set of comparison experiments using the same weight to ensure the same volume, but different areas of (001) facets. In this way, the area of (001) facets of 70 nm nanorods is twice that of the 135 nm ones (illustrated in Fig. 2a). We prepare DNA solution at pH 7 to rule out the influence of hydrino (H^+^) or hydroxyl (OH^–^) in the amounts of DNA conjugated to the particles. After ligand exchange, the amount of DNA is quantified by checking the absorbance intensity at 260 nm (see ESI Fig. S2f†). Fig. 2c shows that the amount of DNA on the 135 nm nanorods is about half that on the 70 nm nanorods, indicating that DNA mainly replaces OAH on the (001) facets.

**Fig. 3:**
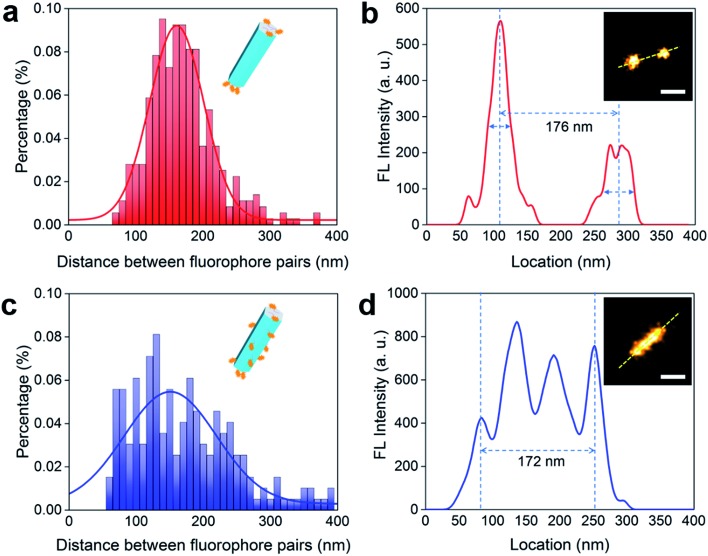
STORM imaging to visualize the facet selective DNA binding to upconversion nanorods. (a and b) Selective binding of DNA molecules on the end (001) facets of UCNP nanorods. (a) Histogram of the distribution of the distance between the two separated fluorescent peaks measured on a batch of upconversion nanorods (170 nm in length). The distance is determined by the method in (b). (b) The fluorescent intensity line profile of a nanorod labelled by a pair of ATTO-550 clusters (inset: STORM image of a nanorod with ends labelled by fluorescent dyes). (c and d) Isotropically modified nanorods display multiple and random fluorescent clusters. (c) Histogram of the distances between two fluorescent peaks for a range of upconversion rods, which are determined according to the method shown in (d). (d) The fluorescent signal profile of a representative nanorod that is isotropically labelled by ATTO-550 dyes (inset: STORM image of the isotropically dye-labelled upconversion nanorod). More STORM results can be found in ESI Section 4.† Scale bar: 100 nm.

In 2016, Su *et al.* employed a super-resolution localization and defocused imaging approach to locate the fluorescent dye molecules on the tips of gold nanorods.^30^ To visualize the selective binding of DNA molecules on the end (001) facets of the UCNP nanorods, we conduct stochastic optical reconstruction microscopy (STORM) to resolve the location of the ATTO-550 labelled DNA molecules conjugated on the UCNP nanorods (170 nm in length, as TEM image shown in ESI Fig. S6†). Consistent to the TEM measurement, the distance between a typical pair of ATTO-550 single molecule clusters is determined to be 176 nm by STORM (Fig. 3b). The Gaussian fit to the histogram distribution of the distance of the pairs of dye clusters reveals a mean value of 170 nm (Fig. 3a), clearly indicating the locations of DNA molecules mainly on the end (001) facets. In contrast, the isotropically modified nanorods display multiple and random fluorescent clusters (Fig. 3d) but with a broader paired distance distribution of ATTO-550 dyes (Fig. 3c). As the controls to confirm that the fluorescent signals on the end of nanorods indeed come from ATTO-550 single molecule fluorophores, the as-synthesized nanorods and non-fluorophore DNA modified nanorods display no fluorescence (see ESI Section 4†).

### Investigate the activity of single strand DNAs on the nanocrystals

**Fig. 4:**
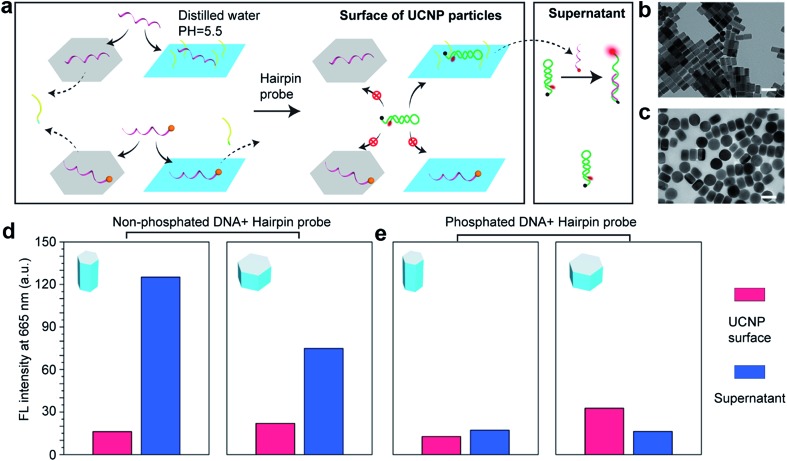
Investigation of the bio activity of single strand DNA on the nanocrystal surface. (a) Illustration of the mechanism: DNA molecules can only replace the OAH on (001) facets of UCNPs but only insert into the OA^–^ molecules on (100)/(010) facets at low pH value; while phosphorylated DNA molecules can replace both OAH and OA^–^ on the surface of UCNPs. It is difficult for the DNA/phosphorylated DNA molecules to hybridize with the hairpin probe on the surface of UCNPs, but inserted DNA molecules would be replaced by hairpin probes and then hybridize with the rest hairpin probes in the supernatant to recover the fluorescent. (b) and (c) are the rod-shape and plate-shape UCNPs used in the experiment. (d) The DNA concentration on the rod-shape UCNPs and plate-shape UCNPs is calibrated to be the same. Larger (100)/(010) area of the rod-shape sample releases more DNA molecules to recover higher fluorescent signals in the supernatant than the plate-shape UCNPs. (e) Phosphorylated DNA molecules bind strongly on both (001) and (100)/(010) facets so very low fluorescent signals are observed for both of the samples. Scale bar: 100 nm.

We employ a hairpin structure DNA^31^ to probe the affinity and activity of single strand DNA on the nanocrystal surface, as shown in Fig. 4. To investigate the anisotropic surface properties for UCNPs, two types of nanocrystals of different aspect ratios (∼55 nm long × ∼30 nm in diameter, and ∼50 nm long × ∼80 nm in diameter) are used. Fig. 4a schematically shows that at pH 5.5, both DNA molecules, with and without phosphate groups on the 5′ terminus, can bind to (001) and (100)/(010) facets of UCNPs. When adding the hairpin probe into the system, phosphorylated DNA molecules strongly bind to the surface of UCNPs, thus no obvious fluorescence singles can be detected either on the nanoparticles sample or the supernatant.

Nevertheless, DNA molecules without phosphate groups can be physically inserted into the hydrophobic OA^–^ surfactants on side surfaces at low pH, although unstable. They can be further released to trigger the hairpin DNA probe to fluoresce. This is verified by the supernatant of rod-shape nanocrystals showing much stronger fluorescent signals than the supernatant of plate-shape nanocrystals. It explains our earlier observation (see ESI Fig. S2b and c†) that low pH values would increase absorption of DNA molecules onto the nanocrystal surfaces, and it is caused by weak physical absorption on the (100)/(010) facets.

### Anisotropic functionalization directed self-assembly of UCNPs

**Fig. 5:**
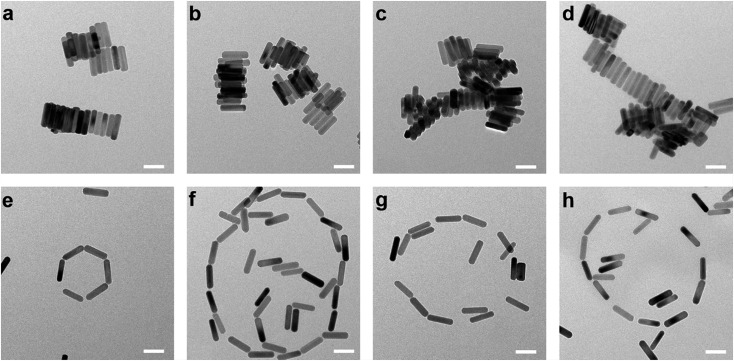
Anisotropic surface functionalization directs the pattern of UCNPs' self-assembly. Side-by-side (a–d) and end-to-end (e–h) pattern of UCNPs self-assembly structure obtained by the anisotropic surfaces and hydrophilic surfaces respectively. Scale bar: 100 nm.

Different self-assembly formats of nanorods can be achieved by controlling the concentration of molecules on the surface.^32^ In this work, the successful control in facet selective functionalization of DNA molecules, either on the (001) facet of UCNPs or on all surfaces of UCNPs, can result in UCNPs with either anisotropic or hydrophilic surface properties. By dispersing the above two kinds of UCNP nanorods (2.5 mg mL^–1^) in water and preparing them onto the copper grid, only after 5 minutes, two self-assembly patterns, side-by-side (Fig. 5a–d) and end-to-end (Fig. 5e–h), can be formed with the efficiencies of 100% and 53.8% respectively. We ascribe these distinct self-assembly behaviours to the theorem of achieving minimum surface energy. DNA molecules are negatively charged because of the existence of phosphodiester bond on the backbone. When the oleic acid molecules of the side facets of UCNPs are not exchanged by the DNA, the side surfaces are inherently hydrophobic. The UCNP nanorods prefer to assemble in a side-by-side manner owing to the mutual attraction between the hydrophobic facets. In contrast, the phosphorylated DNA modified UCNPs have hydrophilic surfaces. The large area of side facets with negatively charged DNAs provides stronger electrostatic repulsion that tends to keep each nanorod away from each other, and the ends with lower energy tend to connect each other forming the end-to-end pattern.

## Conclusion

The key to equipping inorganic nanocrystals with reliable and versatile biomolecular functions lies in the degree of biochemistry control at the bio-/nano-interface, which ultimately determines their stability, specificity, selectivity, and biocompatibility. This work suggests a new dimension in surface modification and functionalization of UCNPs to either have isotropic surface groups or anisotropic surface properties by applying facile DNA ligand exchange method. For the first time, we have shown that we are able to tailor the binding of surface capping ligands based on the facet specific properties of UCNPs, which has been supported by analytical chemistry experiments and super resolution imaging. Our results open a new avenue of selective biomolecule functionalization for nanoscale surface biochemistry, which is beyond the size and morphology controls of nanocrystals. Furthermore, controlled self-assembly of UCNPs enabled by tailored DNA chemistry suggest the promise of using UCNPs as building blocks to construct more sophisticated functionalized nanostructures.

## Methods

### Synthesis of nanocrystals

The nanocrystals were synthesized according to our previously reported method.^27^ Full method regarding the synthesis of the nanocrystals of multiple morphologies are given in ESI.† Briefly, NaYF_4_:Yb,Er nanocrystals were synthesized by thermal solvent method. By tuning the ratio of chemicals, we obtained sphere-like nanocrystals and nanoplates. The nanorods were synthesized by over-growth onto the sphere-like nanocrystals.

### DNA functionalization of nanocrystals by ligand exchange method

Typically, 50 μL of 10 mg mL^–1^ UCNPs cyclohexane suspension was mixed with 400 μL chloroform in a small glass vial. After that 300 μL of 5 μM DNA solution with certain pH values were added to the vial. The UCNPs chloroform suspension and DNA water solution would form two phases. After incubation at 600 rpm on a vortex machine for 3 hours, the UCNPs transferred from chloroform to water phase. It should be noticed that after reaction most of the UCNPs would stay in the interface of water and chloroform if the pH value is high, so all the liquid in the water phase and interface were taken out to centrifuge and the participated nanoparticles were purified by ethanol first to remove the organic solvent and then water. The products were finally suspended in 200 μL distilled water.

### STORM set up, imaging and data analysis

The Stochastic Optical Reconstruction Microscopy (STORM) imaging of UCNPs was carried out with Olympus cellTIRF-4Line system (Olympus IX83 motorized inverted microscope; UPlanSApo TIRF 100 × 1.40 oil; Photometrics EMCCD 512 × 512; CellSens Software; HP Z840 Work Station). After conjugated with ATTO-550 labelled DNA molecules, the UCNPs water suspension was diluted for 1000 times (2.5 × 10^–4^ mg mL^–1^) and a 20 minute ultrasonication was applied before dropping 10 μL into a LabTek 8-well chamber immediately for air-drying. The super-resolution images of ATTO-550 conjugated DNA-oligo labelled UCNPs were acquired at 40 Hz for up to 20 000 frames under the excitation of 561 nm laser (10 kW cm^–2^ at the sample) and activation of 405 nm laser (≤5 kW cm^–2^ at the sample). The excitation beams were reflected by a custom-designed polychroic mirror (z405/488/561/640, Chroma). Fluorescence emissions from ATTO-550 were filtered by a bandpass filter (605/70, Chroma). An imaging buffer (100 mM Tris/HCl pH 8.0, 20 mM NaCl and 10% glucose) with an oxygen scavenger system (60 mg mL^–1^ glucose oxidase, 6 mg mL^–1^ catalase) was used for the STORM imaging. STORM images were analyzed using Insight3 (provided by Dr Bo Huang, UCSF) for single-molecule localization and custom-written Matlab codes for cluster analysis based on nearest-neighbour algorithm.

## Conflicts of interest

There are no conflicts to declare.

## Supplementary Material

Supplementary information
