# Supplementary material for: Anisotropic functionalization of upconversion nanoparticles
Source: Chem Sci. 2018 Apr 23;9(18):4352–8. doi: 10.1039/c8sc01023d (PMC5944387; doi:10.1039/c8sc01023d)
Supplement: Supplementary file 1 [file SC-009-C8SC01023D-s001.pdf]

Electronic Supplementary Material (ESI) for Chemical Science.
This journal is © The Royal Society of Chemistry 2018

Section 1: Synthesis of upconversion nanoparticles (UCNPs)

Reagents:

$YCl_3\cdot 6H_2O$ (99.99%), $GdCl_3\cdot 6H_2O$ (99.99%), $ErCl_3\cdot 6H_2O$ (99.99%), $LuCl_3\cdot 6H_2O$ (99.99%), NH_4F (99.99%), NaOH (99.9%), KOH (99.9%), oleic acid (OA, 90%), and 1-octadecene (ODE, 90%) were purchased from Sigma-Aldrich and used as received without further purification.

Method:

1. Synthesis of $NaYF_4$:Yb,Er core nanocrystals

$NaYF_4$:Yb,Er core nanocrystals were synthesized according to our previously reported method[1]. In a typical experiment, 1 mmol $RECl_3\cdot 6H_2O$ (RE=Y, Yb, Er) with the molar ratio of 78:20:2 were added to a flask containing 6 mL OA and 15 mL ODE. The mixture was heated to 160 °C under argon flow for 30 min to obtain a clear solution and then cooled down to about 50 °C, followed by the addition of 5 mL methanol solution of NH_4F (4 mmol) and NaOH (2.5 mmol). After stirring for 30 min, the solution was heated to 80 °C under argon flow for 20 min to expel methanol, and then the solution was further heated to 310 °C for another 90 min. Finally, the reaction solution was cooled down to room temperature, and products were precipitated by ethanol and washed with cyclohexane, ethanol and methanol for 3 times to obtain the final $NaYF_4$:Yb,Er nanoparticles.

2. Synthesis of $NaYF_4$:Yb,Er nanorods

The longitudinal growth of $NaYF_4$:Yb,Er onto the $NaYF_4$:Yb,Er core nanocrystals was conducted via a successive layer-by-layer hot-injection protocol. Firstly, shell precursors were prepared: 1 mmol $RECl_3\cdot 6H_2O$ (RE=Y, Yb, Er) with the molar ratio of 78:20:2 were added to a 50-mL flask containing 6 mL OA and 15 mL ODE. The mixture was heated to 160 °C under argon flow for 30 min to obtain a clear solution and then cooled down to about 50 °C, followed by the addition of 5 mL methanol solution of NH_4F (4.0 mmol) and NaOH (2.5 mmol) and 3 mL methanol solution of KOH (2.0 mmol). After stirring for 30 min, the solution was heated to 80 °C under argon for 20 min to remove methanol, and then the solution was further heated to 150 °C for another 30 min. Finally, the reaction solution was cooled down to room temperature and labelled as $NaYF_4$:Yb,Er shell precursors.

For the longitudinal growth, 0.2 mmol core particles were added to a 50-mL flask containing 3 mL OA, 7 mL ODE, 69 mg NaOH and 77 mg KOH. The mixture was heated to 160 °C under argon flow for 30 min, and then the solution was further heated to 310 °C. After that, 0.2 mL of the shell precursors were immediately injected into the reaction mixture and ripened at 310 °C for 3 min followed by the same injection and ripening cycles for 50 times to get the nanorods with an average length of 136 nm. The injection and ripening cycles were adjusted to obtain nanorods of various lengths. Finally, the reaction solution was cooled down to room temperature and the formed nanorods were purified according to the procedures used for the purification of $NaYF_4$:Yb,Er core particles.

3. Synthesis of $NaYF_4$:Lu,Yb,Er nanoplates

$NaYF_4$:Lu,Yb,Er nanoplates were synthesized using the method similar to that of $NaYF_4$:Yb,Er core particles. In a typical experiment, 1 mmol $RECl_3 \cdot 6H_2O$ (RE=Y, Lu, Yb, Er) with the molar ratio of 38:40:20:2 were added to a flask containing 6 mL OA and 15 mL ODE. The mixture was heated to 160 °C under argon flow for 30 min to obtain a clear solution and then cooled down to about 50 °C, followed by the addition of 5 mL methanol solution of NH_4F (4 mmol) and NaOH (2.5 mmol). After stirring for 30 min, the solution was heated to 80 °C under argon flow for 20 min to expel methanol, and then the solution was further heated to 310 °C for another 90 min. Finally, the reaction solution was cooled down to room temperature, and products were precipitated by ethanol and washed with cyclohexane, ethanol and methanol for 3 times to get the nanoplates.

Section 2: Computational modelling of the ligands' binding strength

Density-functional theory (DFT) calculations were performed to understand the interaction between the DNA molecules and the β-$NaYF_4$ surfaces. The binding strengths of OAH and OA^- are obtained from our previous publication[1].

DNA-surface adsorption was modelled as an interaction between the terminal functional group of the DNA strand (phosphodiester bonds (PO_2^-)/phosphate group (PO_3^{2-})) and the Y/Na atom on the (001) and (100)/(010) facets of β-$NaYF_4$ nanocrystals. Figure S1 displays the interaction between the functional groups on DNA molecules and the β-$NaYF_4$ surfaces.

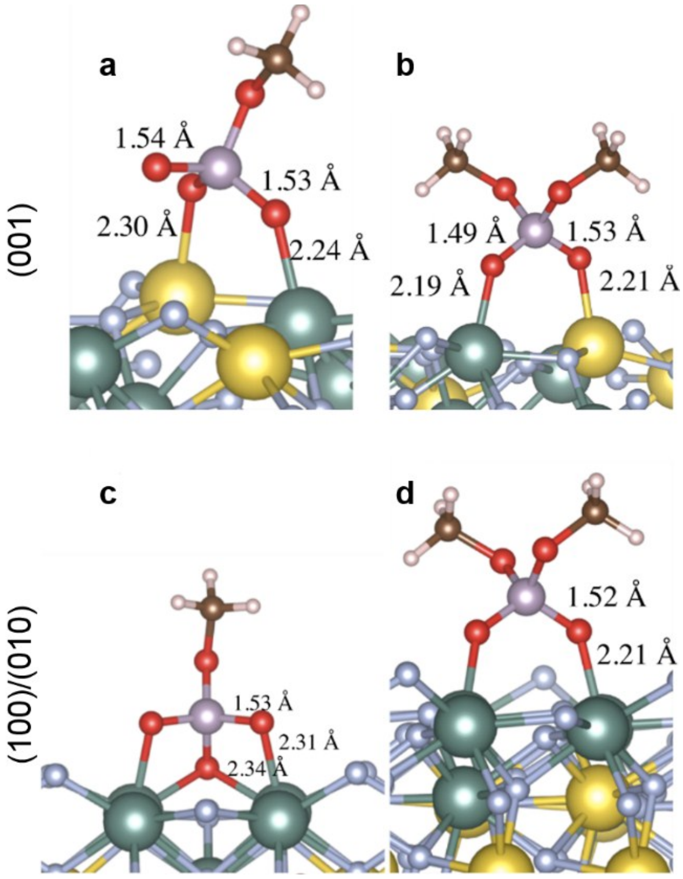

Figure S1. Adsorption configurations for the phosphate group (a and c) and the phosphodiester bond (b and d) considered in our calculations. Purple spheres represent P atoms, red spheres represent O atoms, brown spheres represent C atoms, and small white spheres represent H atoms.

PO_3^{2-} exhibits the strongest binding energy of -57.98 meVÅ^{-2} to (100)/(010) facet, given that it possesses two negatively charged oxygen atoms, and the three oxygen atoms are covalently bonded to two Y atoms. The adsorption energy of OA^- and PO_2^- on the (100)/(010) facet are somewhat close, where OA^- is only 0.44 meVÅ^{-2} weaker than PO_2^- (which represents about 5%

of the adsorption energy of OA^- on (100)/(010)). The difference in adsorption energy between OA^- and PO_2^- is within the error range of DFT and such a minor difference would not initiate the ligand exchange reaction.

On the (001) surface, both PO_2^- and PO_3^{2-} have higher adsorption energy than the original surfactant ligands, which agrees with the experimental observation.

Details of our calculations are provided in below.

Details of the *ab-initio* calculations:

The adsorption of phosphodiester bond and phosphate group on the β-$NaYF_4$ (001) and (100)/(010) facets were calculated in vacuum using spin-unrestricted density functional theory (DFT) within the generalized gradient approximation (GGA) of Perdew, Burke and Ernzerhof (PBE)[2], as implemented in the VASP package, version 5.4.1[3]. The valence electrons are separated from the core by use of projector-augmented wave pseudopotentials (PAW)[4]. The energy cut-off for the plane wave basis set is 500 eV, and the energy tolerance is 10^{-6} to ensure the accuracy of the calculations. The lattice constants of the β-$NaYF_4$ unit cell have been obtained as a=6.0 Å, c=3.61 Å, which is in good agreement with the experimental values of a=5.96 Å, c=3.53 Å[5], and almost identical to the results obtained using the CASTEP code in our previous work[1]. The supercell cell is 10.39 Å×12.0 Å×30.0 Å for the (001) facet and 12.0 Å×14.41 Å×25 Å for the (100)/(010) facet. For the structural energy minimization, the internal coordinates are allowed to relax until all of the forces are less than 0.03 eVÅ^{-1}. The adsorption energy of group G, E_{ad}^{G}, is calculated by using the standard formula

$$E_{ad}^{G} = E_{Surface + G}^{G} - E_{Surface}^{G} - E_{G}$$

where $E_{Surface + G}^{G}$ is the total energy of the group G adsorbed on the surface, $E_{Surface}^{G}$ is the total energy of the pristine surface, and E_{G} is the total energy of the group in vacuum.

Section 3: DNA functionalization of UCNPs by ligand exchange method

Reagents:

Chloroform (99.8%) was provided by ACI Labscan, DNA oligonucleotides were purchased from Integrated DNA Technologies and dissolved in distilled water to 100 μM as stock solution.

Instruments:

TEM characterization of nanoparticles is done by FEI Tecnai T20 Transmission electron microscope. The DNA concentration is evaluated by NANODROP 2000 spectrophotometer, Thermo Fisher Scientific. Zeta potential and size distribution is determined by Malvern Zetasizer Nano-ZS. Horiba iHR 550 spectrograph is used to measure the spectra of upconversion nanoparticles.

Result:

UV-visible absorption profiles show that with the decrease of the PH value of water, more DNA molecules can be absorbed on the surface of UCNPs. Zeta potential and dynamic light scattering results indicate that phosphorylated DNA (P-DNA) capped UCNPs have better stability and smaller size distribution than the ones capped with DNA molecules without phosphate group modification, which further tells phosphate group modification provides DNA molecules stronger binding affinity and higher surface coverage on the UCNPs. After phase transfer from chloroform to water via ligand exchange, there is an obvious decrease of the fluorescence intensity of upconversion nanocrystals. The ratio between the green and the red emission also drops owing to the surface quenching effect[6, 7].

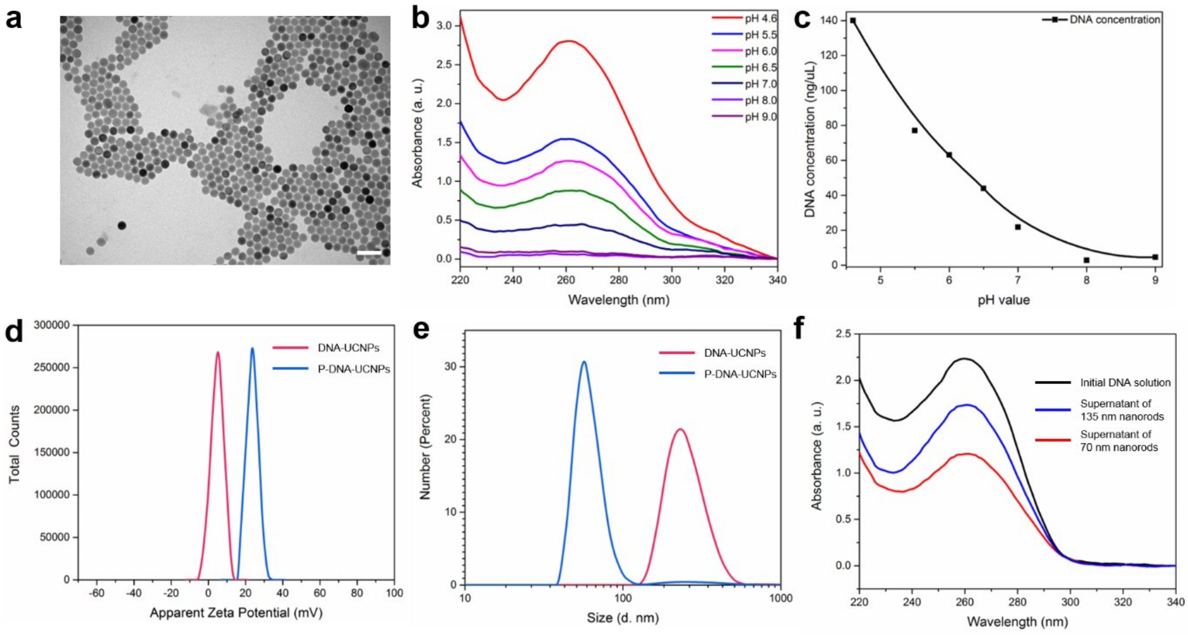

Figure S2. (a) As-synthesized core UCNPs used for the preliminary ligand exchange experiment, scale bar: 100 nm. (b) and (c) show the DNA concentration on the UCNPs after the reaction in water of various pH values. Zeta potential (d) and size distribution (e) of UCNPs conjugated by DNA and P-DNA. (f) Absorption spectra of the initial DNA solution (pH 7.0) and the supernatants after ligand exchange with the same weight of two batches of nanorods of 70 nm and 135 nm in lengths, respectively.

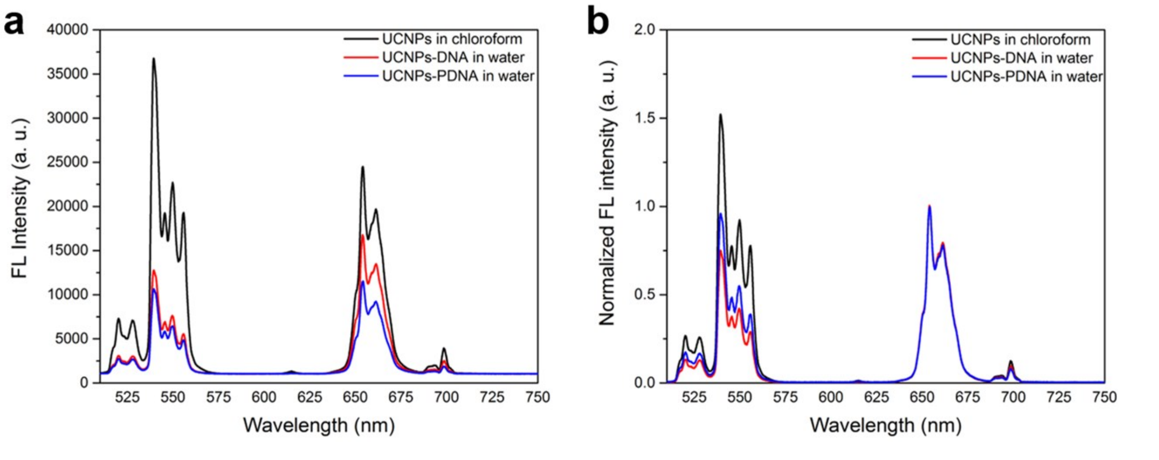

Figure S3. (a) Spectra of as-synthesized UCNPs in chloroform (black line), DNA-modified UCNPs (red line) and phosphorylated DNA-modified UCNPs (blue line) in water. After been transferred from chloroform to water, there is an obvious decrease of the fluorescence intensity owing to the surface quenching effect. (b) Spectra of the UCNPs with different surface modifications normalised against the 650-nm emission peak. The quenching effect on the green band is more significant than that of the red band after being transferred to water phase.

Section 4: STORM images of ATTO-550 fluorophores on nanorods

The listed sequences of DNA oligonucleotides are purchased from Integrated DNA Technologies.

ATTO-550 labelled DNA: 5'/ATTO-550/TTTTTTTTTTTTTTTTTTTT/3'

Non-fluorophore labelled DNA: 5'/TTTTTTTTTTTTTTTTTTTT/3'

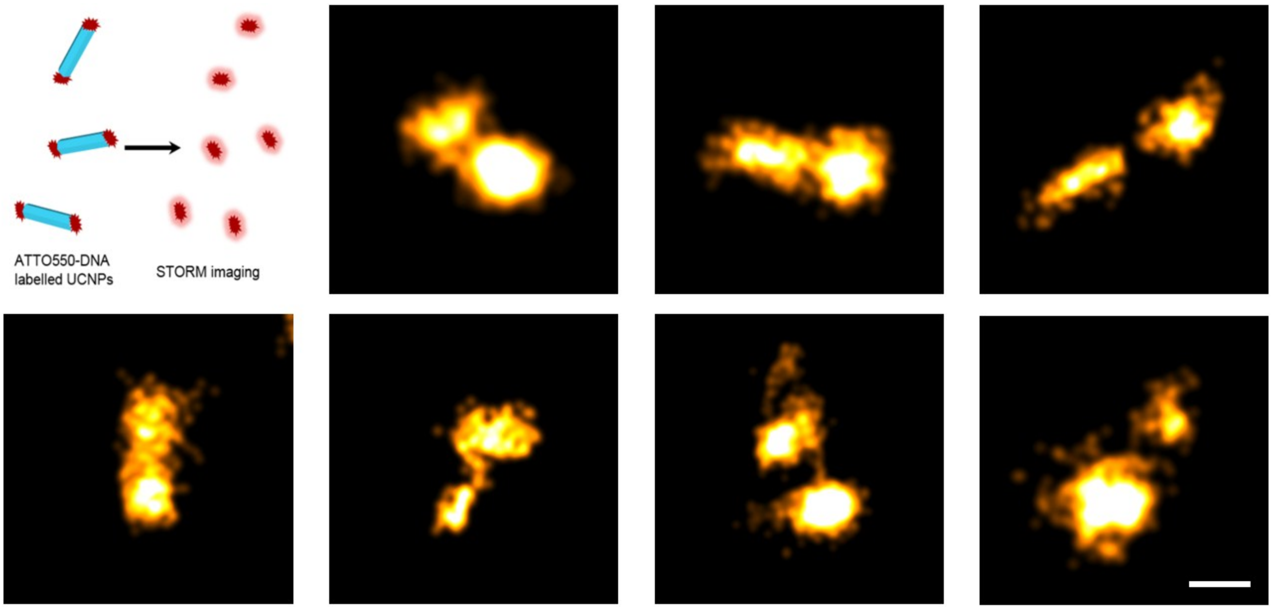

Figure S4. Scheme of STORM imaging to localize ATTO-550 fluorophores on the nanorods and several typical images. Scale bar: 100 nm.

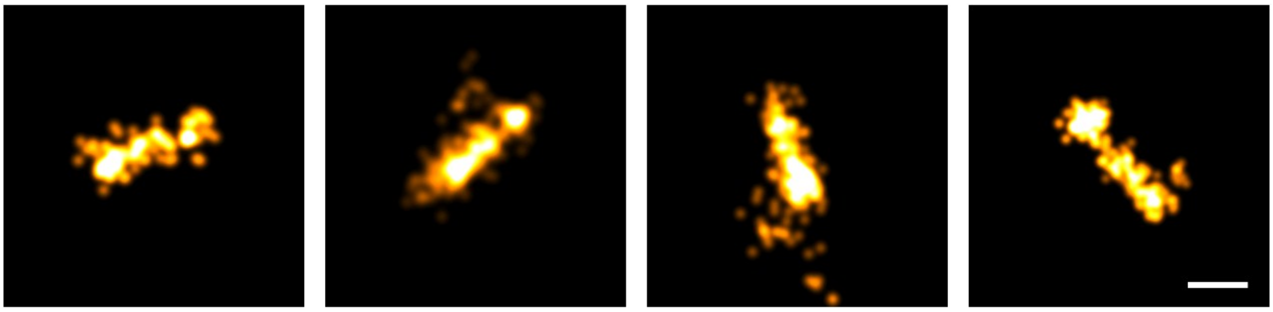

Figure S5. STORM images of fluorescence signals on nanorods isotropically modified with ATTO-550 modified DNA molecules. Scale bar: 100 nm.

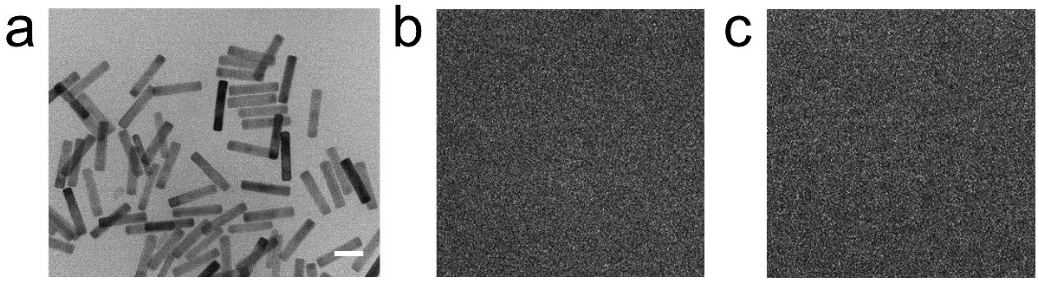

Figure S6. (a) Upconversion nanorods of 170 nm in length used for the STORM experiment. Both as-synthesized nanorods (b) and nanorods modified with non-fluorophore labelled DNA molecules (c) show no signals under the excitation of 561 nm (10 kW/cm^2 at the sample) and no STORM imaging was performed. Scale bar: 100 nm.

Section 5: Test the DNA activity on the nanocrystals

The listed sequences of DNA oligonucleotides in this work are adopted from literature[8] and synthesized by Integrated DNA Technologies.

Hairpin probe:

5'/Cy5/<u>TTAACC</u>CACGCCGAATCCTAGACT**CAAAGT**AGTCTAGGATTCGGCGTG/Quencher/3'

Anchor DNA: 5'/AGTCTAGGATTCGGCGTGGGTTAA/3'

Phosphorylated anchor DNA: 5'/AGTCTAGGATTCGGCGTGGGTTAA/phosphate group/3'

The sticky end of the hairpin probe is underlined and the loop is bolded.

DNA design: The hairpin structure of the probe DNA includes 18-base pair stem, a 6-nucleotide loop and 6-nucleotide sticky end at 5' terminus. Fluorophore cyanine (Cy5) and quencher Iowa Black® RQ are modified on 5' and 3' terminus respectively. After annealing to form the hairpin structure, there are only 6 nucleotides between the Cy5 and the quencher, thus the fluorescence signal is quenched. A 24-nucleotide target single strand DNA is then designed to be complementary to the stem and the loop of the probe DNA, and after hybridization the hairpin structure will open to push the quencher away from the Cy5 to recover the fluorescence.

Anchor DNA and phosphorylated anchor DNA were dissolved in distilled water (pH 5.5) and bound to the UCNPs' surface respectively by the ligand exchange method mentioned before. After the reaction and purification by centrifugation, calibration was done by using UV-visible spectrum to make sure the same amounts of anchor DNA and phosphorylated anchor DNA are used for the hybridization reaction. All the following reactions were carried out in Tris buffer (pH 7.5) with 0.75 M NaCl concentration. Firstly, the hairpin probe was denatured at 95 °C for 3 minutes and annealed by setting at ambient temperature for 2 hours to form the hairpin structure. After that, the hairpin DNA solution was added to the anchor DNA/phosphorylated anchor DNA capped UCNPs suspension for hybridization. The anchor DNA (b*-a) is complementary to the sticky end (b) and the connected stem part (a*) of the hairpin probe. Initially, the Cy5 labelled on the 5' end is quenched by the quencher on the 3' end. Once hybridization occurs, the hairpin structure would open and the Cy5 and the

quencher get apart to recover the fluorescence. Therefore, by monitoring the fluorescence single from Cy5 (Cary Eclipse Fluorescence Spectrophotometer) one can judge if the hybridization occurs or not. We tested the hybridization in solution and it has been proved that only with the existence of anchor DNA the fluorescence single can get recovered.

References

1. D. Liu, X. Xu, Y. Du, X. Qin, Y. Zhang, C. Ma, S. Wen, W. Ren, E. M. Goldys and J. A. Piper, *Nature communications*, 2016, **7**, 10254.
2. J. P. Perdew, K. Burke and M. Ernzerhof, *Physical review letters*, 1996, **77**, 3865.
3. G. Kresse and J. Furthmüller, *Physical review B*, 1996, **54**, 11169.
4. P. E. Blöchl, *Physical review B*, 1994, **50**, 17953.
5. F. Wang, Y. Han, C. S. Lim, Y. Lu, J. Wang, J. Xu, H. Chen, C. Zhang, M. Hong and X. Liu, *nature*, 2010, **463**, 1061.
6. N. Bogdan, F. Vetrone, G. A. Ozin and J. A. Capobianco, *Nano letters*, 2011, **11**, 835-840.
7. F. Wang, J. Wang and X. Liu, *Angewandte Chemie*, 2010, **122**, 7618-7622.
8. R. M. Dirks and N. A. Pierce, *Proceedings of the National Academy of Sciences of the United States of America*, 2004, **101**, 15275-15278.
